# Emerging Viruses in Human Populations

**DOI:** 10.3201/eid1312.070794

**Published:** 2007-12

**Authors:** Aaron C. Brault

**Affiliations:** University of California, Davis, California, USA

**Keywords:** emerging viruses, book review

With increasing international travel and globalization of the world’s economies, changing climates, and altered human behavior and demographics, multiple viruses have emerged to occupy expanded ecologic niches, producing disease syndromes in parts of the world where they had never before existed. Because most emerging viral diseases in humans in the 21st century have been zoonotic, Emerging Viruses in Human Populations focuses on this group of viruses. The resulting overview is a book useful for anyone interested in a diverse group of viral agents that have recently elicited novel disease syndromes in human populations around the world. This text does an excellent job of encompassing a wide variety of contact-transmitted enzootic viruses including severe acute respiratory syndrome–associated coronavirus, Nipah and Hendra viruses, influenza virus, hantaviruses, monkeypox viruses, and vector-transmitted agents including Crimean-Congo hemorrhagic fever, dengue, West Nile, and Japanese encephalitis viruses.

Two especially informative chapters, the first and last, introduce several emerging viral disease agents that affect humans. The authors provide a synthesis of factors that could be associated with the emergence of novel viral agents, such as environmental change, altered human demographics, and human behavior. They also discuss the defining mechanisms through which emerging viral disease can be identified and monitored.

The text outlines basic virologic characterization such as replication strategy and the role of known viral proteins in viral pathogenesis, diagnostics, treatment, and vaccine availability. Additionally, it covers epidemiology of agents, relative disease manifestation, and disease patterns identified in human populations. My only criticism regarding this fine resource is the lack of a consistent level of information presented for each viral agent. In some cases, for example, extensive information was presented on the role of all known viral proteins in replication of the virus and how these proteins contribute to disease manifestations. For other agents, the epidemiology was highlighted with relatively no coverage of viral pathogenesis.

Many of the chapters are easily readable by the general public, yet the level of detail within most of the sections makes this also an excellent reference text for research and public health professionals. I recommend this book for anyone interested in obtaining a broad perspective on the emergence of viral diseases that affect humans.

